# Higher Trait Impulsivity and Altered Frontostriatal Connectivity in Betel-Quid Dependent Individuals

**DOI:** 10.3389/fnhum.2020.578913

**Published:** 2020-10-29

**Authors:** Zhaoxin Qian, Shaohui Liu, Xueling Zhu, Lingyu Kong, Neng Liu, Dongcui Wang, Canhua Jiang, Zhongyuan Zhan, Fulai Yuan

**Affiliations:** ^1^Department of Emergency, Xiangya Hospital, Central South University, Changsha, China; ^2^Health Management Center, Xiangya Hospital, Central South University, Changsha, China; ^3^Department of Radiology, Xiangya Hospital, Central South University, Changsha, China; ^4^Department of Nursing, Xiangya Hospital, Central South University, Changsha, China; ^5^Department of Oral and Maxillofacial Surgery, Xiangya Hospital, Central South University, Changsha, China

**Keywords:** betel quid dependence, trait impulsivity, frontostriatal circuit, PPI, functional connectivity

## Abstract

**Objective**: Betel quid dependency (BQD) is characterized by functional and structural brain alterations. Trait impulsivity may influence substance dependence by impacting its neurobiological underpinnings in the frontostriatal circuit. However, little is known about the trait impulsivity and its neural correlates in individuals with BQD.

**Methods**: Forty-eight participants with BQD and 22 normal controls (NCs) were recruited and scanned on a 3T MRI scanner. Barratt impulsiveness scale (BIS) was used to measure trait impulsivity: motor, attention, and no plan impulsivity. We used voxel-based morphometry (VBM) to assess the relationship between trait impulsivity and gray matter volumes. The relevant clusters identified were served as regions of interest (ROI) seeds. The whole-volume psycho-physiological interactions (PPI) analysis was used to investigate the changes of functional connectivity related to ROI seeds in the cue-reactivity task condition (BQ and control images).

**Results**: Behaviorally, the BQD group showed significantly higher trait impulsivity including motor and no plan impulsivity than the NCs group. VBM analyses showed that motor impulsivity was negatively associated with gray matter volume of right caudate in the whole sample. No difference in gray matter volume between the two groups was observed. PPI analyses showed that there was a significantly decreased functional connectivity between the right caudate and right dorsolateral prefrontal cortex (DLPFC) when watching BQ related images than control images in individuals with BQD. Furthermore, functional connectivity between the right caudate and right DLPFC was negatively correlated with BQ dependency scores.

**Conclusions**: Our study demonstrated the structural basis of trait impulsivity in the caudate and provided evidence for abnormal interactions within frontostriatal circuitsin individuals with BQD, which may provide insight into the selection of potential novel therapeutic targets for the treatment of BQ dependency.

## Introduction

Betel quid (BQ, the product of areca nut, which is the fruit of the areca palm) is among the most widely used psychoactive substances worldwide along with tobacco, alcohol, and caffeine (Boucher and Mannan, 2002; Yen et al., 2018). With a chemical structure analogous to that of nicotine (Lord et al., 2002), BQ has been recognized as a group 1 carcinogen by International Agency for Cancer Research (World Health Organization, 2004), and categorized as “addiction” with heavy use (Mirza et al., 2011; Papke et al., 2015). There are more than 600 million people using BQ within the Indo-Asia-Pacific biogeographic region, and its use is spreading into Asian migrant communities in western countries in recent years (Gupta and Warnakulasuriya, 2002; Lee et al., 2018). In mainland China, BQ is most commonly used in Hunan and Hainan provinces with different eating styles (Zhang and Reichart, 2007). The negative consequences of excessive BQ use have been reported to be correlated with the risk of oral potentially malignant disorder, oral cancer, and other health consequences (Lee et al., 2003; Jacob et al., 2004; Tilakaratne et al., 2006; Mehrtash et al., 2017). Although these clinical phenomena are well-known, the pathophysiological mechanism of BQ dependency (BQD) remains unclear.

Like other psychoactive substances, a high quantity of BQ use is addictive. The research suggested a substantial proportion of BQ users showed signs of dependence, which were associated with the number of chews per day, years of chewing, education, and the inclusion of tobacco in the quid (Benegal et al., 2008; Mirza et al., 2011). A betel quid dependency scale (BQDS) was developed to assess the degree of dependency in BQ users (Herzog et al., 2014), which had been proved with good reliability and validity in our previous studies (Yuan et al., 2017a,b; Zhu et al., 2017, 2018). In a recent study of 8,922 participants across six Asian communities (Taiwan, China, Malaysia, Indonesia, Nepal, and Sri Lanka), betel-quid use disorder was found to meet the Diagnostic and Statistical Manual of Mental Disorders (fifth edition; DSM-V) criteria for a substance use disorder and had a high prevalence among BQ users (Lee et al., 2018).

Functional neuroimaging studies have implicated BQ addiction involves brain structural and functional alterations (Kessler, 2012). Based on recent lines of evidence, individuals with BQ dependency have been documented to be associated with changes in the prefrontal cortex (PFC), insula, anterior cingulate cortex, hippocampal/hypothalamus, cerebellum, frontotemporal and frontoparietal, which are implicated in reward, impulsivity and cognitive systems in the brain (Chen et al., 2015; Liu et al., 2015, 2016a,b; Huang et al., 2017; Yuan et al., 2017a; Zhu et al., 2017; Weng et al., 2018). For example, our previous study suggested heavy BQ users with decreased gray matter volumes in the prefrontal cortex (Zhu et al., 2018), altered white matter integrity in anterior thalamic radiation, and disrupted default mode network connectivity (Zhu et al., 2017). Furthermore, the duration of BQ use and the severity of BQ dependency were reported to be associated with the majority of brain alterations in BQ users. However, the neural mechanism underlying BQD remains largely unclear, and further investigation is needed.

Impulsivity, as a kind of personality trait, is characterized by the propensity to act quickly and without regard for negative consequences (Dalley et al., 2011). Despite the variability in samples and the diversity in measures of impulsivity, the relationship between impulsivity and substance addiction has been widely investigated (Matt et al., 2001; Baker and Yardley, 2002; Shillington and Clapp, 2002). Impulsivity was regarded as one risk factor for the development and maintenance of substance misuse problems, especially in alcohol (Lejuez et al., 2010; Ming et al., 2017), nicotine (Joos et al., 2013), and methamphetamine dependency (Simons and Carey, 2002).

With the development of neuroimaging techniques, the neural bases underlying trait impulsivity have gained much attention in recent literature. Evidence from functional neuroimaging data implies that trait impulsivity may influence substance dependency by impacting its neurobiological underpinnings in the frontostriatal circuit (Knutson et al., 2001; Moreno-López et al., 2012), such as the striatum (caudate and putamen), prefrontal regions, orbitofrontal cortex and anterior cingulate cortex (Horn et al., 2003; Forstmann et al., 2008; Andrews-Hanna et al., 2011; Diekhof et al., 2012). Structural neuroimaging studies have reported the structural manifestation of impulsivity in the prefrontal regions and striatum not only in healthy individuals (Cho et al., 2013; Tschernegg et al., 2015) but also in patients with psychiatric conditions, such as alcoholics (Beck et al., 2009), pathological gambling (Koehler et al., 2015), major depressive disorders (Dombrovski et al., 2012) and psychopathy (Glenn et al., 2010). However, the relationship between impulsivity and the frontostriatal circuit remains unknown in BQ addiction.

To the best of our knowledge, no research has investigated the trait impulsivity deficits and its neural correlates in individuals with BQD. In the current study, our first aim was to measure the characteristics of trait impulsivity by using the Barratt impulsiveness scale (BIS) in the BQD group (Patton et al., 1995). The second goal was to examine the structural manifestation of trait impulsivity by investigating the association of trait impulsivity and gray matter volumes. Then, the relevant areas identified were served as regions of interest (ROI) seeds. A whole-volume psycho-physiological interactions (PPI) analysis was conducted to investigate the changes of functional connectivity related to ROI seeds in the cue-reactivity task condition (BQ and control images). We hypothesized that compared with healthy controls, individuals with BQD showed abnormal impulsivity, which would correlate with the frontostriatal circuit. The results of this study could help to understand the underlying psychological and neural bases of BQ use, and further have potential implications for treating and preventing BQ dependency.

## Materials and Methods

### Participants

Participants (*N* = 70, all males) were recruited from Changsha, Hunan province. We recruited two groups of participants: BQD group (*N* = 48) and normal controls (NCs) group (*N* = 22). Individuals with BQD were recruited from the outpatient department in Xiangya Hospital of Central South University. Structured Clinical Interview was used to determine if the BQD group met the DSM-V criteria for substance use disorders (average scored on 7.63 ± 1.70). The NCs group were recruited through a combination of targeted site sampling, advertisement, and snowball sampling referrals.

All participants were screened with the Structured Clinical Interview for DSM-IV Axis I disorders and were excluded for any of the following: past or current Axis I disorder, including but not limited to major depressive disorder and/or any anxiety disorders; current or past other substance use; pregnancy or current breastfeeding; unstable medical or neurological illness; the history of severe head trauma; and the presence of metal implants precluding a magnetic resonance imaging (MRI) scan. Additionally, none of the participants was diagnosed as alcohol or smoking dependency as measured by the AUDIT (Alcohol Use Disorders Identification Test) and the FTND (Fagerstrom Test for Nicotine Dependence) respectively. Diagnosis and exclusion criteria were corroborated by two licensed psychiatrists.

A detailed history of BQ use was identified for everyone with BQD: age of first BQ use, duration (years) of BQ use, and estimated BQ use per day (g). This study only included males because there was significantly less problem with BQ use in females (Lee et al., 2018). The study was approved by the Institutional Review Board at Xiangya Hospital of Central South University. All participants provided written informed consent after the study procedures were explained to them thoroughly.

### Procedures

All participants were asked to come to Xiangya Hospital and finish the behavior measures and MRI scans. They got compensation for their time devoting to this study (on average about 1-h interview and 30 min MRI scan). All of them were required to be abstinent (4 h) from tobacco, alcohol, and caffeine drinking before the interview. The MRI scans included a high-resolution structural scan and a session of fMRI scan with the cue-reactivity task. The cue reactivity task was a frequently used task to investigate the brain functional mechanism of individuals with addiction (Kühn and Gallinat, 2011).

### Behavior Measures

#### Barratt Impulsiveness Scale (BIS)

The BIS is a self-report questionnaire designed to assess the personality/behavioral construct of impulsiveness, which has been widely used in impulsivity research for the last 50 years (Patton et al., 1995). As a 30-item rated on a five-point scale, it includes three subscales: motor, attention, and no plan subscales. The motor scale comprises items that reflect acting without thinking. The attention scale includes items measuring poor concentration/attentiveness with those reflecting cognitive instability. The no plan scale measures an orientation focused on the immediate present that fails to consider future effects. The BIS exhibited high degrees of reliability and validity in both English and Chinese version (Yao et al., 2007; Huang et al., 2013). The Chinese version of the BIS used in this current study exhibited high internal consistency, with a Cronbach’s alpha value of 0.81, 0.78, and 0.83 for three subscales.

#### Betel Quid Dependence Scale (BQDS)

The BQDS is a widely used scale for diagnosing the dependency of BQ (Lee et al., 2012). As a 16-item self-report instrument, the BQDS comprises of three factors: “physical and psychological urgent need,” “increasing dose,” and “maladaptive use.” The BQDS was found to have good internal consistency (alpha = 0.92) and construct validity, which exhibited high degrees of reliability and validity in both the English-speaking and Chinese-speaking chewers (Herzog et al., 2014; Zhu et al., 2017).

### MRI Scans

All MRI images were acquired by using a Siemens 3.0T Prisma scanner at Xiangya Hospital. Standard settings were used to perform the scan. For example, foam pads were used to minimize head motion. Participants were instructed to keep their head very still during the structural scan and respond to the instructions when doing functional scans. Stimulus presentation, the timing of all stimuli, and response events were achieved by using Matlab (Mathworks) and Psychtoolbox^1^ on an IBM-compatible PC. Participants’ responses were collected online using an MRI-compatible button box.

The structural scan was performed using T1 MPRAGE sequence, covering the whole brain with the following scanning parameters: TR/TE = 2,110 ms/3.18 ms, matrix = 256 × 256, number of slices = 256, and voxel size = 0.7 × 0.7 × 0.7 mm^3^, sagittal slice position. The functional scan was performed using EPI sequence with the following parameters: TR/TE = 2,000 ms/30 ms, matrix = 64 × 64, number of slices = 75, voxel size = 2.34 × 2.34 × 2.00 mm^3^.

### Cue Reactivity Task

**Figure 1:**
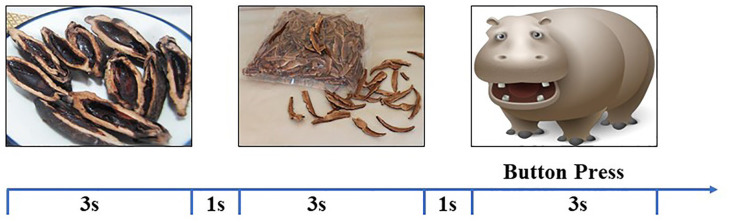
The illustration of stimulus presentation. Different types of images [Betel quid (BQ), control, and animal] were randomly presented for 3 s with 1 s interstimulus interval. Participants were instructed to press a button when seeing an animal image.

Participants performed one session of the cue reactivity task inside the scanner. During this task, two types of cues were presented: the BQ and control images. There were 20 images for each category, and each image was presented three times. To keep all participants awake during the passive view task, 10 animal images were presented twice (see Figure 1). These images were presented with a random order with each image for 3 s with a 1-s intertrial interval. Participants were instructed to press a button whenever they saw an animal. The correct ratio and reaction in the cueing task were served as the behavior data of the task.

### Data Analysis Procedure

Behavioral measures were first compared between two groups to find the differences of demographic as well as the impulsivity measure. Then significant trait impulsivity characteristics were correlated with structural MRI data in the whole sample to find their anatomical bounds. The structural MRI data were analyzed by the voxel-based morphometry (VBM) method, which has been widely used in neuroimaging studies (Ashburner and Friston, 2000; Good et al., 2001; He et al., 2013). Significant clusters in the VBM analysis were served as ROI seeds to do the PPI analysis. The PPI maps were then compared between the two groups. All tests were corrected for multiple comparisons with Bonferroni correction.

### VBM Analysis

VBM analysis was implemented in FSL_VBM (Smith et al., 2004), which has been widely used to analyze the structural MRI data. The processing steps were standardized: brains were extracted by using BET (Smith, 2002) and segmented into gray matter, white matter, and CSF by using FAST4 (Zhang et al., 2001). Two steps of registration (linear and non-linear) were performed to register the gray-matter partial volume images to the standard space (MNI152). A study-specific template was created by averaging all normalized images. Lastly, the resulting images of gray matter volume were smoothed with an isotropic Gaussian kernel (3 mm). Statistics were performed with FSL non-parametric permutation methods (Randomise v2.1; Nichols and Holmes, 2002). Statistical analysis using the general linear model was used to identify the correlation between gray matter volumes and trait impulsivity. The null distribution at each voxel was constructed using 10,000 random permutations. Multiple comparisons were corrected across the whole brain using the threshold-free cluster enhancement (TFCE). Additionally, we also performed the analysis to compare the difference of gray matter volume between the BQD and NCs group.

### fMRI Data Analysis

fMRI data preprocessing and statistical analyses were carried out by FSL^2^. Images were realigned to compensate for small residual head movements (Jenkinson and Smith, 2001). Translational movement parameters never exceeded one voxel in any direction for any participant. Data were spatially smoothed using a five-mm full-width-half-maximum (FWHM) Gaussian kernel and were filtered using a nonlinear high pass filter with a 100-s cutoff. A two-step registration procedure was used whereby EPI images were first registered to the MPRAGE structural image, and then into standard MNI space, using affine transformations (Jenkinson and Smith, 2001). Registration from MPRAGE structural image to standard space was further refined using FNIRT nonlinear registration (Andersson et al., 2007a,b).

Statistical analyses were performed in the native image space, with the statistical maps normalized to the standard space before higher-level analyses. The data was modeled at the first-level using a general linear model within FSL’s FILM module. As illustrated before Andersson et al. (2007a,b), brain activations were modeled for BQ, control, and animal images separately. The event onsets were convolved with canonical hemodynamic response function (HRF, double-gamma) to generate regressors. Temporal derivatives were included as covariates of no interest to improve statistical sensitivity. The six-movement parameters were also included as covariates in the model.

### PPI Analysis

PPI analysis was performed by FSL^3^. Two interaction terms of BQ and control images with the ROIs were entered into the model. Group analyses were performed to examine group differences between interactions and to specifically find the difference in brain connectivity in BQD and NCs groups. Group images were evaluated with a height threshold of *Z* > 3.1 and a cluster probability of *p* < 0.05, corrected for whole-brain multiple comparisons based on Gaussian random field theory. The education was included as a covariate for all fMRI analyses.

## Results

### Demographic Results

Table 1 showed the demographic and impulsivity characteristics for all participants. According to Table 1, the BQD and NCs group were matched on age (BQD: 34.85 ± 8.10 years; NCs: 32.05 ± 6.25 years; *t*_(68)_ = 1.44, *p* = 0.15), and BMI (BQD: 25.07 ± 3.74 kg/m^2^; NCs: 23.32 ± 3.84 kg/m^2^; *t*_(68)_ = 1.81, *p* = 0.08). However, they did show significant difference on years of education (BQD: 11.62 ± 2.83 years; NCs: 17.82 ± 2.82 years; *t*_(68)_ = −6.19, *p* < 0.001). Years of education was entered as a covariate for the following analysis. Table 1 illustrated the difference of trait impulsivity between the two groups. Results suggested that the BQD group showed higher motor and no plan impulsivity than the NCs group. Both groups performed very well in the cue-reactivity task (over 97% of the correct ratio of detecting animals). There was no significant difference in terms of either the correct ratio or reaction time between groups.

**Table 1:** Demographic and impulsivity characteristics of participants (M ± SD).

	BQD	NCs	Statistics
*N*	48	22	-
Age (years)	34.85 ± 8.10	32.05 ± 6.25	*t*_(68)_ = 1.44, *p* = 0.150
Education (years)	11.62 ± 2.83	17.82 ± 2.82	*t*_(68)_ = −6.19, *p* < 0.001*
BMI (kg/m^2^)	25.07 ± 3.74	23.32 ± 3.84	*t*_(68)_ = 1.81, *p* = 0.080
BQDS	59.63 ± 14.55	-	-
Duration of BQ use (years)	15.23 ± 7.10	-	-
Age of first BQ use	17.13 ± 6.67	-	-
Estimated BQ use per day (g)	40.19 ± 33.11	-	-
BIS_Motor	25.52 ± 5.86	20.59 ± 4.29	*t*_(68)_ = 3.53, *p* = 0.001*
BIS_Attention	36.83 ± 4.13	37.73 ± 4.31	*t*_(68)_ = −0.83, *p* = 0.410
BIS_No Plan	39.27 ± 4.72	36.73 ± 3.55	*t*_(68)_ = 3.06, *p* = 0.003*
Cue_CR	0.99 ± 0.03	0.97 ± 0.05	*t*_(68)_ = 1.77, *p* = 0.160
Cue_RT (ms)	609.5 ± 138.1	618.8 ± 144.3	*t*_(68)_ = −0.25, *p* = 0.800

*Significant at *p* < 0.05.

### VBM Results

**Figure 2:**
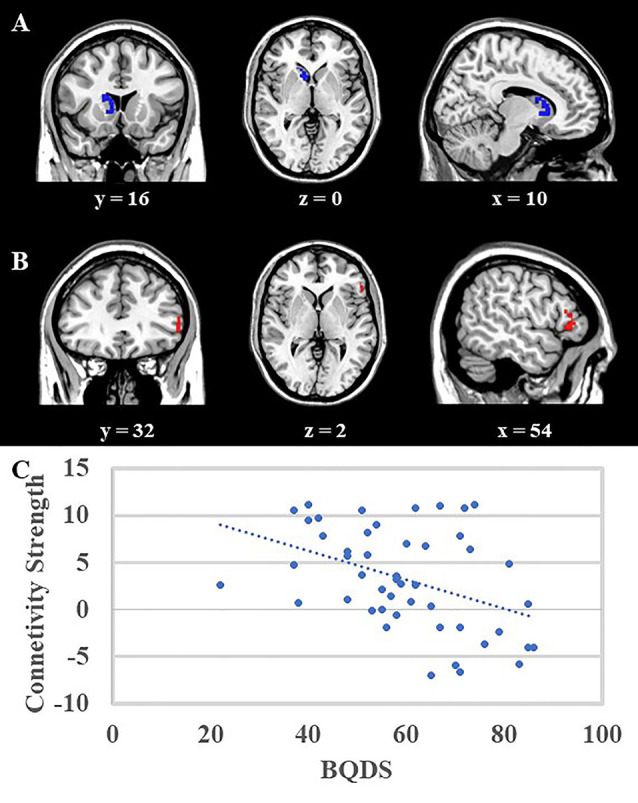
**(A)** Voxel-Based Morphometry (VBM) analysis showed that the right caudate (blue area, MNI coordinates: 8, 14, −2; 81 voxels, *Z* = 4.74) was negatively correlated with scores of motor impulsivity, and no other results were found. **(B)** Psycho-Physiological Interactions (PPI) analysis suggested that in contrast to view control images, viewing BQ related images would decrease functional connectivity between right caudate and right DLPFC (red area, MNI coordinates: 54, 28, −2, 57 voxels, *Z* = 4.38). Both results were mapped onto a standard brain and displayed with coronal, axial, and sagittal views respectively. The numbers below represented the slice numbers. **(C)** The scatter plot of correlation analysis between functional connectivity (right caudate and right DLPFC) and BQDS scores in the BQD group.

VBM correlation analysis aimed to investigate the structural bonding of the trait impulsivity characteristics. The result of correlation analysis suggested that scores of motor impulsivity were negatively correlated with the volume of right caudate in the whole group (both BQD and NCs group; Figure 2A, right caudate, blue area, MNI coordinates: 8, 14, −2; 81 voxels, *Z* = 4.74). Moreover, the right caudate volume was negatively correlated with BQDS scores in the BQD group (*r*_(48)_ = −0.441, *p* = 0.002). Also, no significant difference was found between cognitive impulsivity, no plan impulsivity, and gray matter volumes between BQD and NCs group.

### PPI Results

The right caudate which showed a significant correlation with motor impulsivity was used as the ROI to do the PPI analysis. PPI results suggested that in contrast to viewing control images, viewing BQ related images would decrease functional connectivity between the right caudate and right dorsolateral prefrontal cortex (DLPFC) in the BQD group (Figure 2B, DLPFC, red area, MNI coordinates: 54, 28, −2; 57 voxels, *Z* = 4.38). Furthermore, correlation analysis showed that functional connectivity between right caudate and right DLPFC was negatively correlated with BQDS scores in the BQD group (Figure 2C, *r*_(48)_ = −0.417, *p* = 0.003).

## Discussion

To our knowledge, the current study provided the first empirical evidence to demonstrate the behavioral and neural differences of trait impulsivity between the BQD and NCs group. Consistent with our hypothesis, the BQD group showed significantly higher motor impulsivity and no plan impulsivity than the NCs group. Motor impulsivity was negatively associated with gray matter volume in right caudate in the whole group. Compared with the NCs group, the BQD group showed less functional connectivity between right caudate and right DLPFC when viewing BQ related images than control images, which was more profound in participants with higher BQDS score.

This current study highlighted the trait impulsivity deficit in the BQD group. Substance misusers have been widely reported to score higher on measures of trait impulsivity despite a variety of measures of impulsivity (Dawe et al., 2004; Crews and Boettiger, 2009). So far, evidence has been accumulated for the relationship between impulsive behaviors and substance use and abuse (Baker and Yardley, 2002; de Wit, 2009). One study found that high trait impulsivity predicted the switch to compulsive cocaine-taking (Belin et al., 2008). Another study detected a positive relationship between the frequency of marijuana use and the number of marijuana-related problems that were greatest in those with high trait impulsivity scores (Simons and Carey, 2002). Similar evidence also came from internet addiction (Cao et al., 2007), sexual addiction (Bancroft and Vukadinovic, 2004), as well as other substance addiction (Kreek et al., 2005). Our study extends these prior findings by providing new evidence for increased motor and no plan impulsivity in individuals with BQ dependency.

The present study provided evidence for a negative correlation between gray matter volume in right caudate and motor impulsivity, which supported previous reports on the structural manifestation of trait impulsivity in the striatum (including caudate and putamen). In healthy individuals, a negative relationship was revealed between impulsivity scores assessed by BIS and gray matter volumes of the putamen bilaterally (Cho et al., 2013). In psychiatric disorders, Babbs et al. (2013) reported a negative correlation between trait impulsivity and caudate activity in response to a milkshake in the overweight group. Other studies suggested that structural and functional asymmetry of the caudate was associated with impulsivity deficits in participants with attention-deficit/hyperactivity disorder (Schrimsher et al., 2002; Dang et al., 2016). Although reports on gray matter changes reflecting high impulsivity in the striatum were inconsistent (Glenn et al., 2010), our results provide new evidence for a negative relationship between trait impulsivity and right caudate by the results that higher motor impulsivity was associated with less right caudate volume and further support the notion that the right caudate is involved in the pathophysiology of trait impulsivity in substance addiction.

Finally, this current study revealed decreased functional connectivity between right caudate and DLPFC in individuals with BQD (viewing BQ related images vs. control images). Anatomically, the dorsal striatum (caudate and putamen) receives projections primarily from the association cortex (mainly DLPFC), sensory, and motor areas (Alexander et al., 1986). Neuroimaging studies in addiction reveal the critical roles of the frontostriatal circuit, which are mainly associated with reward (striatum) and cognitive control (prefrontal cortex; Noël et al., 2013; He et al., 2017, 2019; Wei et al., 2017; Chen et al., 2018). It is worth noting that the striatum and prefrontal cortex are intermodulations by frontostriatal circuits (Volkow et al., 2011, 2012; Saad et al., 2019). The interactions between the striatum and prefrontal cortex are especially important to investigate the underlying neural mechanism of addiction, such as smoking (Kober et al., 2010) and internet gaming disorder (Yuan K. et al., 2017; Kim and Kang, 2018). Previous studies revealed structural and functional alterations in the prefrontal cortex and caudate in individuals with BQD relative to healthy controls (Liu et al., 2016b; Sariah et al., 2019). However, the interaction between the prefrontal cortex and caudate in BQ users with dependency has never been investigated. Our findings contribute to filling this gap by showing that reduced functional connectivity between right caudate and right DLPFC in individuals with BQD. Furthermore, it should be noted that functional connectivity between right caudate and DLPFC was revealed to be negatively associated severity of BQ dependency, which implies the effect of BQ use on the frontostriatal circuits.

It was important to note some of the limitations of this study. Firstly, the study used an imbalanced sample. Future research involving a balanced sample and larger sample sizes may help address some of these additional questions. Secondly, this study only recruited male participants, so it should be cautious in generalizing the findings of this study to the females. Then, the cross-sectional nature of this study can’t make causal association conclusions about BQ use and impulsivity. Longitudinal studies should be employed in the future to assess the longer-term effect of BQ use on impulsivity in individuals with BQD. Lastly, we also screened some participants with symptoms of depression and/or anxiety. BQ use may be associated with higher depression or anxiety. We will carry out future studies to investigate this topic.

In conclusion, we revealed increased motor and no plan impulsivity in individuals with BQD relative to healthy controls. Motor impulsivity was negatively associated with gray matter volume of right caudate in the whole group. Compared with the NCs group, the BQD group showed less functional connectivity between right caudate and right DLPFC when viewing BQ related images than control images, which was more profound in participants with higher BQDS scores. Our study sheds light on the pathology of trait impulsivity in individuals with BQD, which may provide insight into the selection of key targeted brain regions for interventions aiming to decrease motor impulsivity levels in betel-quid chewers.

## Data Availability Statement

The raw data supporting the conclusions of this article will be made available by the authors, without undue reservation.

## Ethics Statement

The studies involving human participants were reviewed and approved by the Institutional Review Board at Xiangya Hospital of Central South University. The patients/participants provided their written informed consent to participate in this study.

## Author Contributions

FY, ZQ, and SL conceived and designed the experiments. SL, NL, and CJ conducted the experiments and collected data. DW and ZZ analyzed the results. ZQ, SL, XZ, and LK wrote the main manuscript text. All authors reviewed the manuscript. All authors contributed to the article and approved the submitted version.

## Conflict of Interest

The authors declare that the research was conducted in the absence of any commercial or financial relationships that could be construed as a potential conflict of interest.

## Footnotes

^1^www.psychtoolbox.org

^2^www.fmrib.ox.ac.uk/fsl

^3^https://fsl.fmrib.ox.ac.uk/fsl/fslwiki/PPI
